# Genetic heterogeneity and mutational signature in Chinese Epstein-Barr virus-positive diffuse large B-cell lymphoma

**DOI:** 10.1371/journal.pone.0201546

**Published:** 2018-08-14

**Authors:** Fang Liu, Zhe Wang, Xiaoge Zhou, Qing Liu, Gang Chen, Hualiang Xiao, Weihua Yin, Shigeo Nakamura, Huilan Rao

**Affiliations:** 1 Department of Pathology, Foshan Hospital, Sun Yat-sen University, Foshan, Guangdong Province, China; 2 State Key Laboratory of Cancer Biology, Department of Pathology, Xijing Hospital, Fourth Military Medical University, Xi'an, Shannxi Province, China; 3 Department of Pathology, Beijing Friendship Hospital, Capital Medical University, Beijing, China; 4 Department of Pathology, Fujian province Cancer Hospital, Fuzhou, Fujian Province, China; 5 Daping Hospital, Army Medical University, Chongqing, China; 6 Department of Pathology, Shenzhen Hospital, Peking University, Shenzhen, Guangdong Province, China; 7 Department of Pathology and Clinical Laboratories, Nagoya University Hospital, Nagoya, Japan; 8 Department of Pathology, Sun Yat-sen University Cancer Center; State Key Laboratory of Oncology in South China; Collaborative Innovation Center for Cancer Medicine, Guangzhou, Guangdong Province, China; German Cancer Research Center (DKFZ), GERMANY

## Abstract

Epstein-Barr virus (EBV)-positive diffuse large B-cell lymphoma (EBV+ DLBCL) is typically an aggressive tumor in elderly patients. However, in a subset of young patients, EBV+ DLBCL follows a relatively indolent clinical course and exhibits a good response to chemotherapy. This lymphoma comprises polymorphous lymphoma and large cell lymphomas subtypes, with the latter subtype showing a significantly poorer prognosis. It is unknown whether the genetic background differs between age groups and histopathological subtypes. To investigate the genetic basis, heterogeneity, and recurrently mutated genes in EBV+ DLBCL, we performed whole-exome sequencing of DNA from 11 tissue samples of this lymphoma. Sequencing revealed that the most common substitution was the transition C>T/G>A. Genetic features—including the numbers of mutated genes in exonic region, single-nucleotide variants (SNV), and indels—did not significantly differ between age groups or histological subtypes. Matching with the COSMIC database revealed that the main mutational signature was signature 3, which is associated with failure of DNA double-strand break-repair by homologous recombination. Mutant-Allele Tumor Heterogeneity (MATH) scores showed that EBV+ DLBCL exhibited broad intratumor heterogeneity, and were positively correlated with Ann Arbor Stage and ≥2 extranodal lesion sites. We identified 57 selected recurrently mutated genes. The most commonly mutated five genes—*LNP1* (11/11), *PRSS3* (10/11), *MUC3A* (9/11), *FADS6* (9/11), and *TRAK1* (8/11)—were validated by Sanger sequencing. These mutated genes have not previously been identified. Overall, our present results demonstrate the tremendous genetic heterogeneity underlying EBV+ DLBCLs, and highlight the need for personalized therapeutic approaches to treating these patients.

## Introduction

Epstein-Barr virus (EBV)–positive diffuse large B-cell lymphoma (EBV+ DLBCL, NOS) [1] constitutes 3–11% of all DLBCLs. EBV+ DLBCL can occur in patients of any age, but elderly and young patients typically differ with regards to preferential lesion sites, morphological features, and prognosis. In elderly patients, EBV+ DLBCL usually exhibits an aggressive clinical course with a median survival of 2 years or early relapse despite treatment with strong chemotherapy [2], while young patients typically present with nodal disease and better survival [3]. Hong et al. [4] propose that EBV+ DLBCL in young patients might be a clinically distinct disease entity from EBV+ DLBCL of the elderly, likely involving mechanisms of lymphomagenesis other than immunosenescence. EBV+ DLBCL comprises polymorphous lymphoma (PL) and large cell lymphomas (LCL) subtypes, with a prominent inflammatory microenvironment and some degree of geographic necrosis. Compared to PL, the LCL subtype shows a significantly poorer prognosis [5].

EBV+ DLBCL is characterized by prominent classical and alternative NF-_k_B activation [6]. EBV latent membrane protein 1 (LMP1)-positive large tumor cells reportedly produce CCL17 and CCL12, and exhibit accumulation of CCR4-expressing cells, including regulatory T cells [7]. Kato et al. [8] performed gene set enrichment and gene ontology analysis, and found enrichment of the Janus kinase signal transducer and activator of transcription-related genes (JAK/STAT) pathway and NF-_k_B pathways. However, few studies report genetic data. Translocation analysis involving single-locus genes reveals that translocations of *Myc*, *BCL6*, or *IgH* genes are less common in EBV+ DLBCL than in EBV− DLBCL [9]. Yoon H et al. [10] analyzed copy number alterations (CNAs) and gene profiles, and found that EBV+ DLBCL harbored fewer genomic alterations compared to EBV− DLBCL [10]. To date, the genomic profile of EBV+ DLBCL is poorly defined, and it is unclear whether the genetic background of this lymphoma differs between age groups and histopathological subtypes.

Whole-exome sequencing (WES) can be used to examine a genetic spectrum and heterogeneity, identify somatic mutations that contribute to lymphomagenesis, predict prognosis, and identify novel therapeutic targets. In the present study, we performed WES of 11 cases of EBV+ DLBCL, and further investigated mutated genes in an expanded patient series, with the aim of elucidating the genetic heterogeneity and mutational signature of EBV+ DLBCL.

## Materials and methods

### Patient samples

Tissue samples were obtained from 11 patients at the time of their diagnosis with EBV+DLBCL between 2014 and 2017. Table 1 presents the detailed clinical characteristics of the included patients. The 11 tissue samples were evaluated, and then DNA was extracted from formalin-fixed, paraffin-embedded (FFPE) samples.

**Table 1:** Clinicobiological features of the 11 EBV+ DLBCL patients analyzed by whole-exome sequencing (WES).

NO.	Age/ gender	Pathological subtype	Lesion sites	B symptoms	Ann Arbor Stage	LDH (>ULN)	β2-Microglobulin (>ULN)	IPI Risk	Proliferation index (%, Ki67)	Therapy	Response	Status (‡OS, month)
1	67/F	**PL**	**Multiple LN + tonsil + heart septa**	yes	**IIIB**	**yes**	**NA**	**NA**	**70%**	6*R-EPOCH	**PD**	NA
2	65/M	LCL	Left tonsil + Multiple LN + bone marrow	yes	**IV**	**-**	**yes**	**high**	**90%**	7*R-CHOP	**CR**	AWD (19+)
3	68/F	PL	**left cervical** LN	yes	**II**	**NA**	**yes**	**NA**	**50%**	1*CHOP	**PD**	AWD (17+)
4	79/M	LCL	**left cervical** LN	yes	**NA**	**NA**	**NA**	**NA**	**50%**	**No treatment**	**NA**	**DOD(19)**
5	55/M	LCL	**Multiple LN**	yes	**NA**	**NA**	**no**	**NA**	**80%**	**NA**	**PD**	AWD (31+)
6	24/F	PL	**left cervical LN + root of tongue**	no	**II**	**yes**	**no**	**low**	**95%**	6*CHOP	**CR**	AWD (38+)
7	28/M	LCL	**right cervical** LN + liver + spleen + **humerus + femur**	yes	**IVB**	**no**	**NA**	**low**	** NA**	2*R-CHOP	**CR**	AWD (87+)
8	52/M	PL	Bilateral inguinal LN	no	**IV**	**-**	**no**	**low**	**40%**	3*R-CHOP	**CR**	AWD (27+)
9	31/M	LCL	**left cervical LN** + liver + **pleuro + peritoneum**	no	**IIIB**	**yes**	**NA**	**low**	**80%**	4*R-CHOP	**PD**	AWD (18+)
10	61/M	LCL	Right tonsil + LN	yes	**II**	**yes**	**yes**	**low**	**NA**	3*R-CHOP	**PD**	DOD (4)
11	66/M	LCL	left groin LN	**NA**	**II**	**NA**	**NA**	**NA**	**80%**	**NA**	**NA**	DOD (13)

Abbreviations: EBV+ DLBCL, Epstein-Barr virus-positive diffuse large B-cell lymphoma; F, female; M, male; PL, polymorphous lymphoma; LCL, large cell lymphoma; LN, lymphoma node; NA, not available; R-EPOCH, doxorubicin, vincristine, etoposide; PD, progressive disease; AWD, alive with disease; DOD, died of disease; CHOP, cyclophosphamide, doxorubicin, vincristine, and prednisone; R-CHOP, rituximab, cyclophosphamide, doxorubicin, vincristine, and prednisone; CR, complete response; PD, progressive disease; IPI, International Prognostic Index; LDH, lactate dehydrogenase; ULN, upper level of normal; WES, whole-exome sequencing.

‡OS from sampling.

EBV+ DLBCL was diagnosed according to the 2016 WHO classification of lymphoid neoplasms [1]. EBV was detected using an *in situ* hybridization ISH technique with the EBV ISH kit (Leica Microsystems, Wetzlar, Germany). This study only included cases of EBV+ DLBCL that showed nuclear positivity for the EBV-encoded small RNA (EBER) in a majority (>50%) of neoplastic cells. We excluded EBV+ DLBCL cases in the clinical setting of known immune deficiency.

Informed consent was obtained from the legal guardians of all participating patients before this study, and we had access to information that could identify individual patients during/after data collection. This study was approved by the Ethics Committee of the Institutional Review Board at Sun Yat-sen University Foshan Hospital [File number: L (2014) NO.2].

### Whole-exome sequencing

Pathologic review of slides stained with hematoxylin and eosin revealed tumor purity ranging from 40–90%. We extracted DNA from the 11 FFPE tissue samples using the QIAamp DNA FFEP Tissue kit, and measured DNA concentrations using PicoGreen dsDNA Quantitation Reagent (Invitrogen). DNA sample quality was evaluated by gel electrophoresis. From 1.5 μg of tumor DNA, we constructed capture libraries using the Agilent SureSelect XT Human All Exon V5 kit. The initial library concentration was detected using a Qubit 2.0 fluorometer, and was adjusted to 1 ng/μl by dilution. The concentration was validated by quantitative polymerase chain reaction (Q-PCR), to ensure a library concentration of >2 nM for sequencing. Next, the enriched exome libraries were multiplexed and sequenced using an Illumina HiSeq PE 150 to generate 150-bp paired-end reads. Exome sequencing was performed to a sufficient depth to achieve a minimum coverage of 15 reads in at least 80% of the coding sequence from the UCSC hg19 transcripts database.

### Sequencing data analysis

First, the Illumina sequencing adapters were removed from the raw reads of each sample using the Trimmomatic program [11], which reported the longest high-quality stretch of each read. Next, the low-quality bases were trimmed from both read ends, using the parameters LEADING:3 TRAILING:3 SLIDINGWINDOW:4:15. Reads of less than 36 bp were dropped. The remaining clean reads were aligned to the hg19 reference genome using Novoalign software version 2.07.14 (http://www.novocraft.com) with BWA [12]. Duplicate reads having paired ends aligning to the same start locations due to optical or PCR artifacts were removed from further analysis using Picard (http://broadinstitute.github.io/picard/). We computed the read coverage and depth based on the final BAM file, and the reads with over 10× depth were considered more credible. Mapped reads with mapping quality scores of at least 20 were used as input for GATK Haplotype Caller [13] to call genome variants. Finally, the called variants were annotated using ANNOVAR.

Matched normal samples were not available for all of the sequenced tumor samples. Therefore, we performed extensive data filtering to remove known SNPs that are reported in databases—including the 1000 Genomes Project that includes variants from various ethnic populations, including Han Chinese. Additionally, we removed any variant that appeared in three ANNOVAR [14] datasets, including 1000g2015aug_all, snp138, and exac03nontcga, to identify the final tumor mutations. The SNP locus in the dbSNP database was removed, but the alterations in the COSMIC database were kept.

### Signatures and tumor heterogeneity analyses

During its lifetime, each individual tumor cell within a human body acquires a certain number of somatic mutations. The catalog of somatic mutations from a tumor genome bears the signatures of the mutational processes that have occurred during tumor development. These mutations originate from a wide spectrum of both endogenous and exogenous mutational processes that generate distinct patterns of mutations, termed mutational signatures, embedded within the cell genome. DeconstructSigs [15] determines the composition of a set of mutational signatures within individual tumor specimens. It can also detect mutational processes active in only a small number of samples and investigate well-established signatures without requiring a large sample set. Furthermore, this approach can evaluate how the activity of mutational processes changes in individual tumors over time.

Given the limited number of tumor samples in this study, we used deconstructSigs to establish the contributions of individual mutational signatures to the samples. This enabled elucidation of the potential contributions of mutational processes within single tumor samples, identification of the weights of known mutational signatures across tumors, and application of those signatures to the samples to determine the contribution of each mutational process to each individual sample.

Tumor genetic heterogeneity is of prime interest for basic and clinical research. Malignant clones evolve under selective pressures, particularly those imposed by therapeutic drugs. To evaluate intratumor genetic heterogeneity, we used the Mutant-Allele Tumor Heterogeneity (MATH) score [16], which effectively describes the spread in the data, to quantify differences in the dispersion or spread of allele frequencies of the tumor samples. MATH scores were calculated based on the distributional differences of mutant-allele frequencies among mutated loci. The scores described the ratio of the width of the data to the center of the distribution [MATH = 100 * median absolute deviation (MAD)/median], providing a quantitative measure of the degree of heterogeneity in a tumor sample, with a heterogeneous tumor having a higher score. Rajput et al. [17] modified this approach, using data from a sequencing panel (Ion Ampliseq Comprehensive Cancer Panel) and analyzing only the tumor samples, which included all heterozygous variants. To compare MATH scores between tumors with high and low heterogeneity, we used a cut-off value of 30 MATH units, based on the small number of samples. We also used non-parametric Wilcoxon rank-sum test to analyze the influence of MATH scores on clinical variables, such as extranodal site (≥2), Ann Arbor stage, proliferation index, therapy response, and prognosis.

### Sanger sequencing validation

Sanger sequencing was performed to analyze the point mutations in five genes—*LNP1*, *PRSS3*, *MUC3A*, *FADS6*, and *TRAK1*—in our 11 EBV+ DLBCL samples and in an additional 16 EBV+ DLBCL samples from other hospitals. All alterations were confirmed. PCR and sequencing primers were designed using NCBI/Primer BLAST, and are presented in S1 Table. The resulting PCR products were purified using the Qiaquick PCR purification kit (Qiagen, Hilden, Germany) and sequenced at Eurofins MWG Operon (Ebersberg, Germany). At least 50 ng of DNA was PCR amplified using 2× HotStar Master Mix (Qiagen, 203443) and 300 nM of each primer. We utilized a touchdown PCR method, with reactions incubated at 94°C for 5 min; followed by 35 cycles of 94°C for 30 s, 60°C for 30 s, and 72°C for 30 sec; and a final extension at 72°C for 5 min. The amplified fragments were verified by agarose gel electrophoresis, and purified using Agencourt Ampure XP beads according to the manufacturer’s instructions (Beckman Coulter Genomics, A63881).

### Statistical analyses

Overall survival (OS) was defined as the period between pathological diagnosis and disease-related death. Associations between categorical variables were assessed using the non-parametric Wilcoxon rank-sum test. All *P* values are two-sided, with the type I error rate fixed at 0.05. All data were analyzed using SPSS software (version 17.0; SPSS Inc., Chicago, IL, USA).

## Results

### Patients characteristics

Table 1 presents the clinical characteristics of 11 patients with EBV+ DLBCL. Among these 11 patients, 5 (45%) had only lymphadenopathies without extranodal involvement, and the remaining 6 (55%) had lymphadenopathies with extranodal lesions. The sites of extranodal involvement included tonsil (n = 3), liver (n = 2), heart septa and spleen, humerus and femur, root of tongue, pleuro, peritoneum, and bone marrow. Of the 11 patients, 3 were under 50 years of age, all of whom were in the International Prognosis Index (IPI) risk group L/LI. Regarding histological subtypes, 4 cases were deemed polymorphous lymphoma (PL), and the remaining 7 were large cell lymphoma (LCL). Most patients received rituximab, cyclophosphamide, doxorubicin, vincristine, and prednisone (R-CHOP) chemotherapy. Regarding prognosis, follow-up duration ranged from 4 to 89 months, and 3 patients died of lymphoma. Table 2 summarizes the patients’ characteristics at diagnosis of EBV+ DLBCL. The histopathological subtype groups significantly differed in gender distribution. But there were no other significant differences in clinical features between the age groups and histopathological subtype groups.

**Table 2:** Patient characteristics at diagnosis of EBV+ DLBCL (11 cases).

Variables	<50 years old (n = 3)	≥50 years old (n = 8)	Polymorphous lymphoma subtype (n = 4)	Large cell lymphoma subtype (n = 7)	P*	*P*^§^
Years of age, median (range)	28 (24–31)	65.5 (52–79)	59.5 (24–68)	61 (28–79)	1.0000	0.0189
Sex, male/female	2/1	6/2	3/1	7/0	0.0242	1.0000
B symptoms presence, %	33% (1/3)	85.7%% (6/7)	50% (2/4)	83.3% (5/6)	0.5000	0.1833
Involved lesion site					1.0000	0.1818
Nodal, %	0% (0/3)	63%(5/8)	50% (2/4)	43% (3/7)		
Nodal + Extranodal, %	100% (3/3)	38% (3/8)	50% (2/4)	57% (4/7)		
Extranodal, %	-	-	-	-		
IPI, %					1.0000	1.0000
L/LI, %	100% (3/3)	80% (4/5)	100% (4/4)	75% (3/4)		
HI/H, %	-	20% (1/5)	-	25% (1/4)		
Therapy					0.1901	0.3173
None	-	-	-	-		
CHOP	33% (1/3)	28.6% (2/7)	50% (2/4)	-		
RCHOP	67% (2/3)	57% (4/7)	25% (1/4)	100% (3/3)		
Other scheme	-	14.3% (1/7)	25% (1/4)	-		
Response to therapy					1.0000	0.5238
CR, %	67% (2/3)	28.6% (2/7)	50% (2/4)	33.3% (2/6)		
PR, %	-	-	-	-		
PD, %	33% (1/3)	71.4% (5/7)	50% (2/4)	67.7% (4/6)		
Relapse, %	33% (1/3)	71.4% (5/7)	25% (1/4)	33.3% (2/6)	1.0000	0.5238
2-year survival, %	100%(3/3)	57.1%(4/7)	100%(3/3)	42.9%(3/7)	1.0000	1.0000

Abbreviations: EBV+ DLBCL, Epstein-Barr virus-positive diffuse large B-cell lymphoma; LDH, lactate dehydrogenase; IPI, international prognostic index; L, low; LI, low-intermediate; HI, high-intermediate; H, high; CHOP, cyclophosphamide, adriamycin, vincristine, prednisone; R-CHOP, rituximab, cyclophosphamide, adriamycin, vincristine, prednisone; CR, complete response; PR, partial response, and PD, progressive disease. Values indicate the number of positive/tested cases.

*Polymorphous lymphoma subtype versus large cell subtype group.

^*§*^<50 years old versus ≥50 years old group.

### Mutation spectrum detected by WES in EBV+ DLBCL

**Fig 1:**
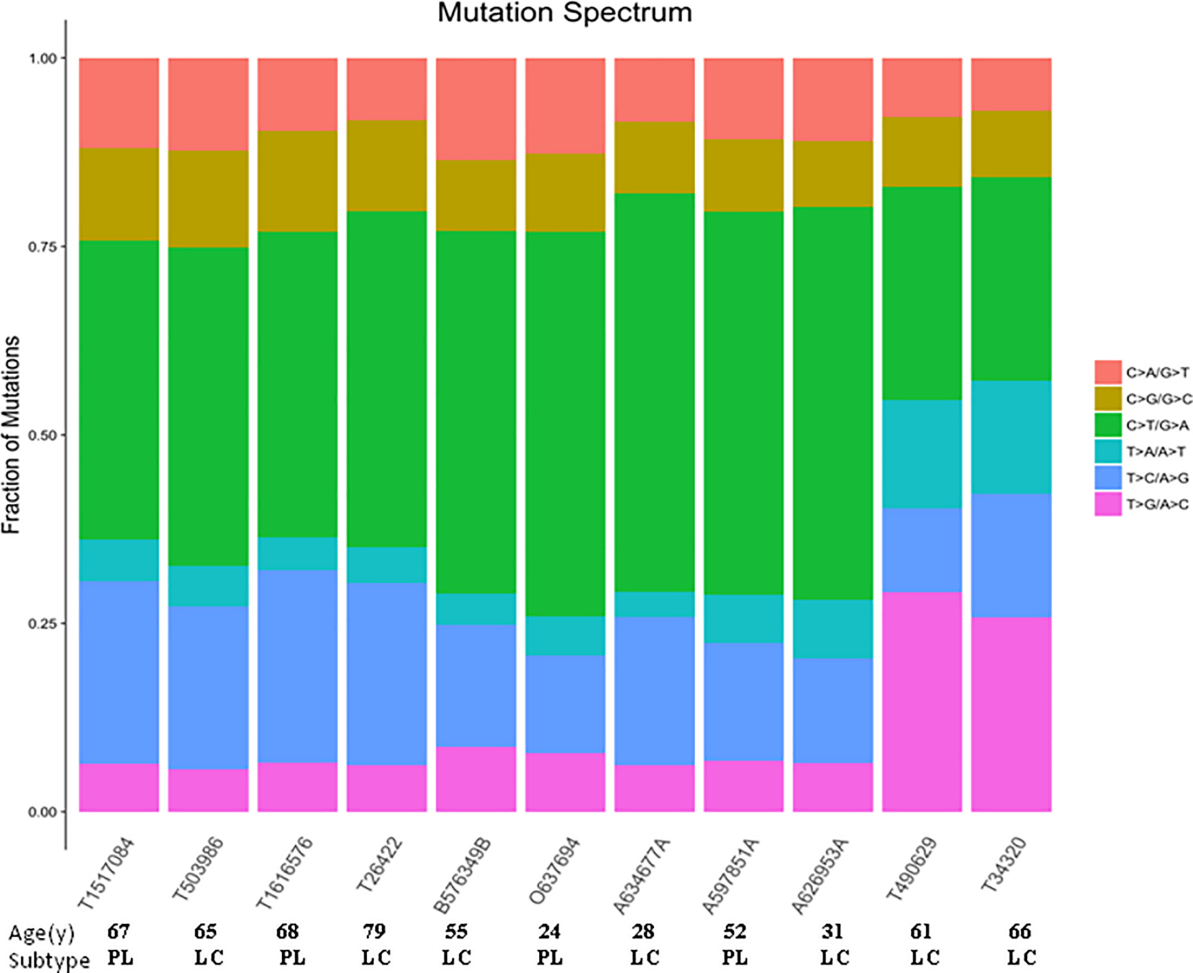
Mutation spectrum of Epstein-Barr virus (EBV)-positive diffuse large B-cell lymphoma shown by whole-exome sequencing. Frequency of substitutions in each sample for the six possible classes of mutation. The most common substitution was the transition C>T/G>A, followed by T>C/A>G.

**Fig 2:**
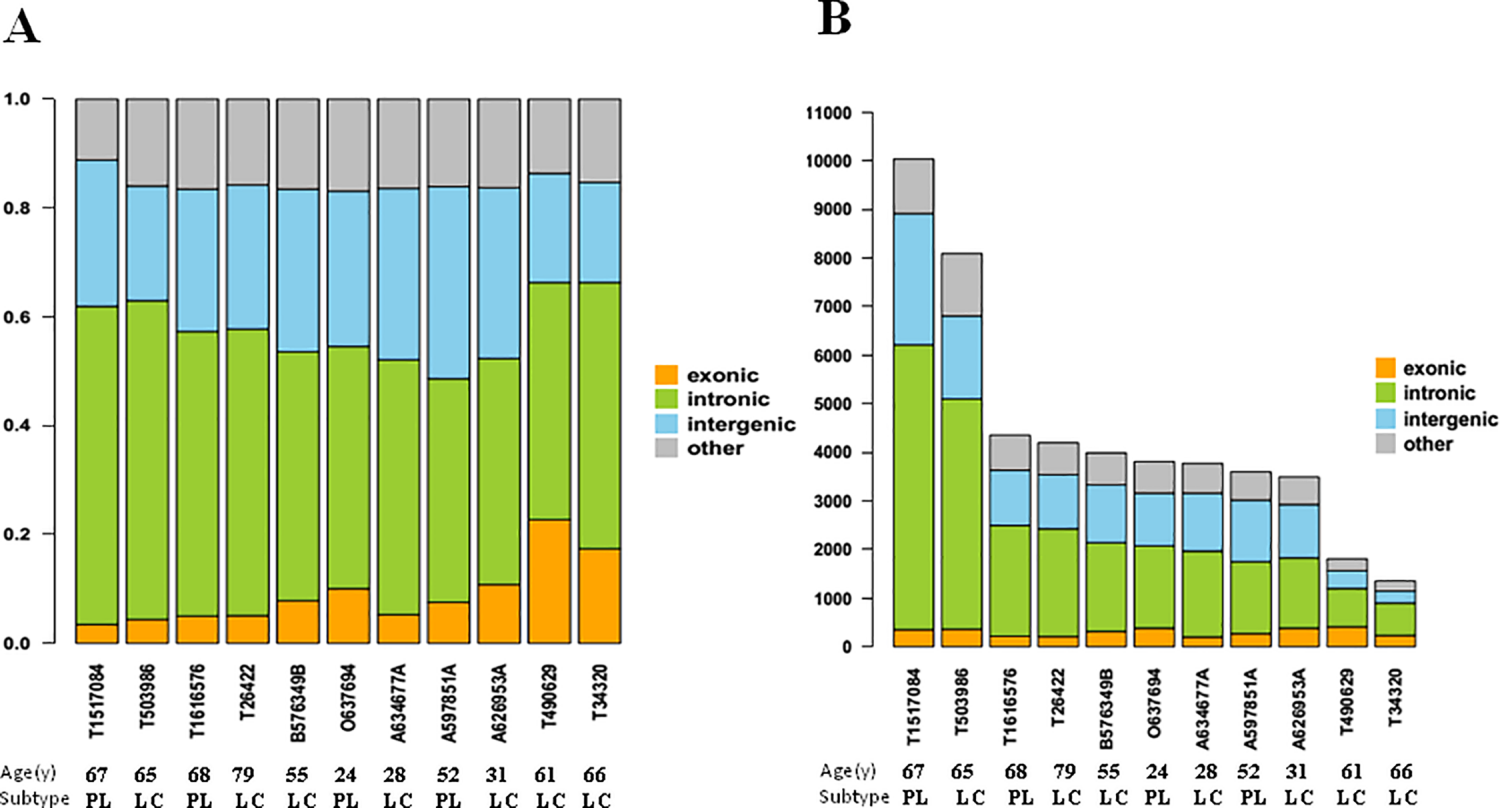
Distribution of mutations in different genome regions of Epstein-Barr virus (EBV)-positive diffuse large B-cell lymphoma detected by whole-exome sequencing. (A) The percentages of mutations in different regions of EBV+ DLBCL cases. The percentage of mutations in exonic regions ranged from 3.49%–22.68% among these 11 cases. (B) We identified 3326 protein-coding genes with somatic mutations affecting exonic regions, with a range from 200 to 410 per case.

Using WES, we characterized the spectrum of mutations in 11 EBV+ DLBCL cases. On average, we generated 75,134,319 high-quality reads per sample to a mean depth of 141-fold exon coverage, with an average of 69% of bases covered per patient (range, 57–78%) (S1 Table). The most common substitution was the transition C>T/G>A (Fig 1). The patient-specific mutation rate was not correlated with the frequency of any particular mutation types. Similarly, the observed mutation rate was nor correlated with the average allelic fraction of those mutations observed in each patient. Among our 11 cases, the percentage of mutations in exonic regions ranged from 3.49%–22.68% (Fig 2A and S2 Table). We identified 3326 protein-coding genes with somatic mutations that affected the encoded protein’s structure (nonsynonymous changes, frameshifts in the coding sequence, synonymous SNVs, and mutations affecting canonical splicing sites), with a median of 302 mutations per case (range, 200–410) (Fig 2B and S2 Table). In total, we identified 57 selected distinct candidate variants in the 11 cases (Fig 2B and S3 Table). Genetic features did not significantly differ between the ≤50 year old and >50 year old groups, or between the PL and LCL subtype groups (Table 3). Notably, the number of variants in all 11 EBV+ DBLCL cases was likely overestimated due to a lack of corresponding germline DNA for WES. All data can be viewed in NCBI(https://www.ncbi.nlm.nih.gov/bioproject/482899)or NODE (http://www.biosino.org/node/project/detail/OEP000144).

**Table 3:** Comparison of the numbers of mutated genes in exonic regions, and ratio to total mutations detected by WES in 11 EBV+ DLBCL cases according to age and pathological subtype.

Groups	Variables	Exonic		(%) Exonic/total	
		mean ± SD	*P* value	mean ± SD	*P* value
Pathological subtype	PL subtype (n = 4)	305.25 ± 74.33	0.925	0.07 ± 0.03	0.299
	LCL subtype (n = 7)	300.71± 85.13		0.10 ± 0.07	
Age	<50 years old (n = 3)	320.00 ± 103.94	0.760	0.09 ± 0.03	0.475
	≥50 years old (n = 8)	395.75 ± 72.92		0.09 ± 0.07	

Abbreviations: EBV+ DLBCL, Epstein-Barr virus-positive diffuse large B-cell lymphoma; WES, whole-exome sequencing; PL, polymorphous lymphoma; LCL, large cell lymphoma.

Note. Values are mean ± SD. *P* ≤ 0.05, non-parametric Wilcoxon rank-sum test was performed.

### Single-nucleotide variants (SNVs) and indels shown by WES

**Fig 3:**
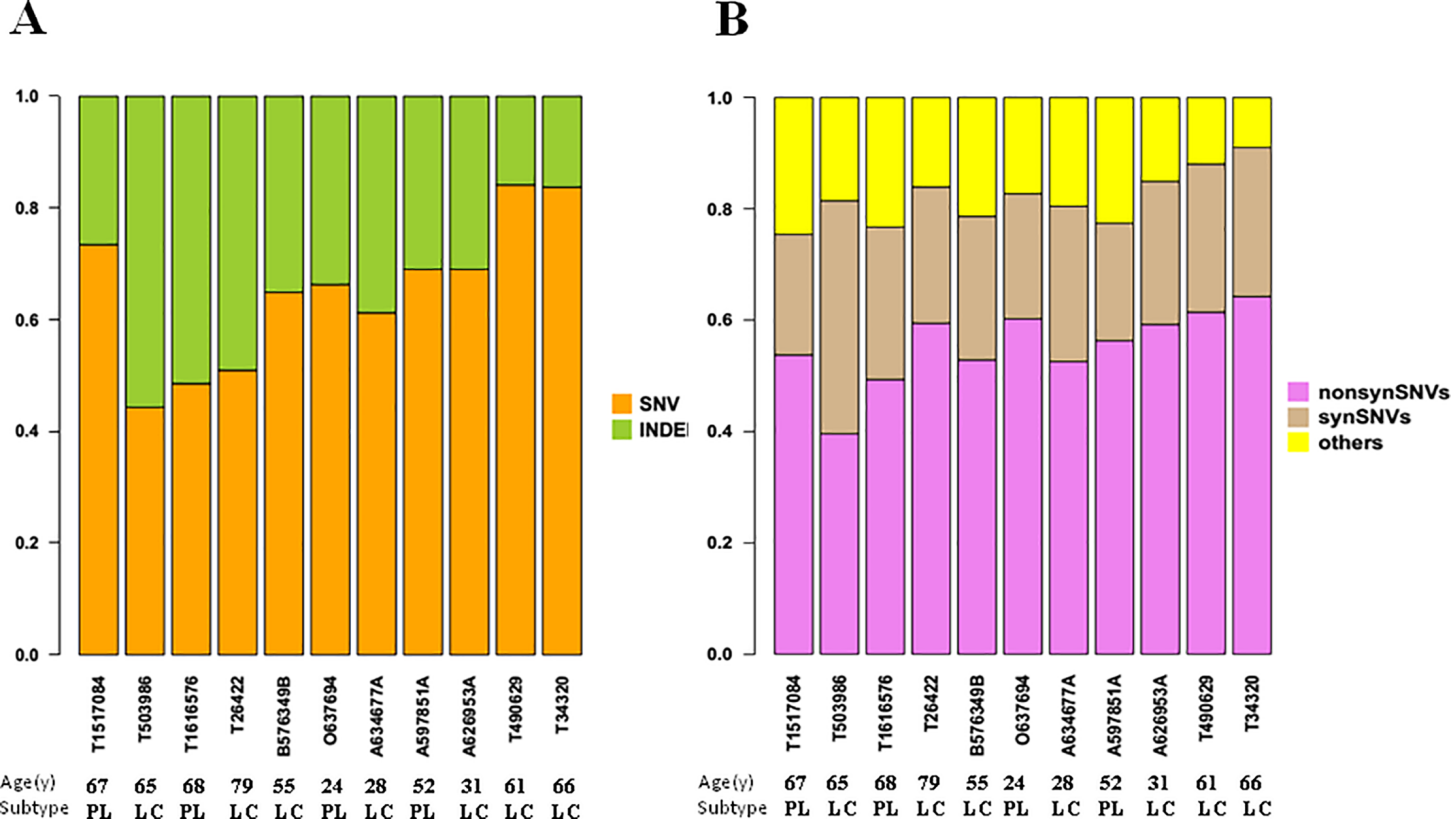
An overview of the data analysis strategy for single-nucleotide variations (SNVs) and indels shown by whole-exome sequencing. On average, we identified 220 (range, 126–358) somatically acquired point mutations per case. Of these, an average of 168 (range, 105–252) were non-synonymous and 81 (range, 52–149) were synonymous. Thus, the ratio of non-synonymous to synonymous was 2.08 (0.95–2.67) mutations per Mb.

Fig 3 presents an overview of our data analysis strategy. Across 11 EBV+ DLBCL samples, we detected an average of 2428 SNVs/case (range, 1132–7379) and 1670 indels/case (range, 220–4517) (Fig 3A and S4 Table). We focused on the somatic SNVs identified in tumor DNA (Fig 3B), finding an average of 220 somatically acquired point mutations/case (range, 126–358). Of these, 168 (105–252) were non-synonymous and 81 (52–149) were synonymous. Thus, the ratio of non-synonymous to synonymous SNVs was 2.08 (0.95–2.67) mutations per Mb (S4 Table). The numbers of SNVs and indels did not significantly differ between age groups or subtype groups (S5 Table).

### Signatures and intratumor clonal heterogeneity of EBV+ DLBCL

**Fig 4:**
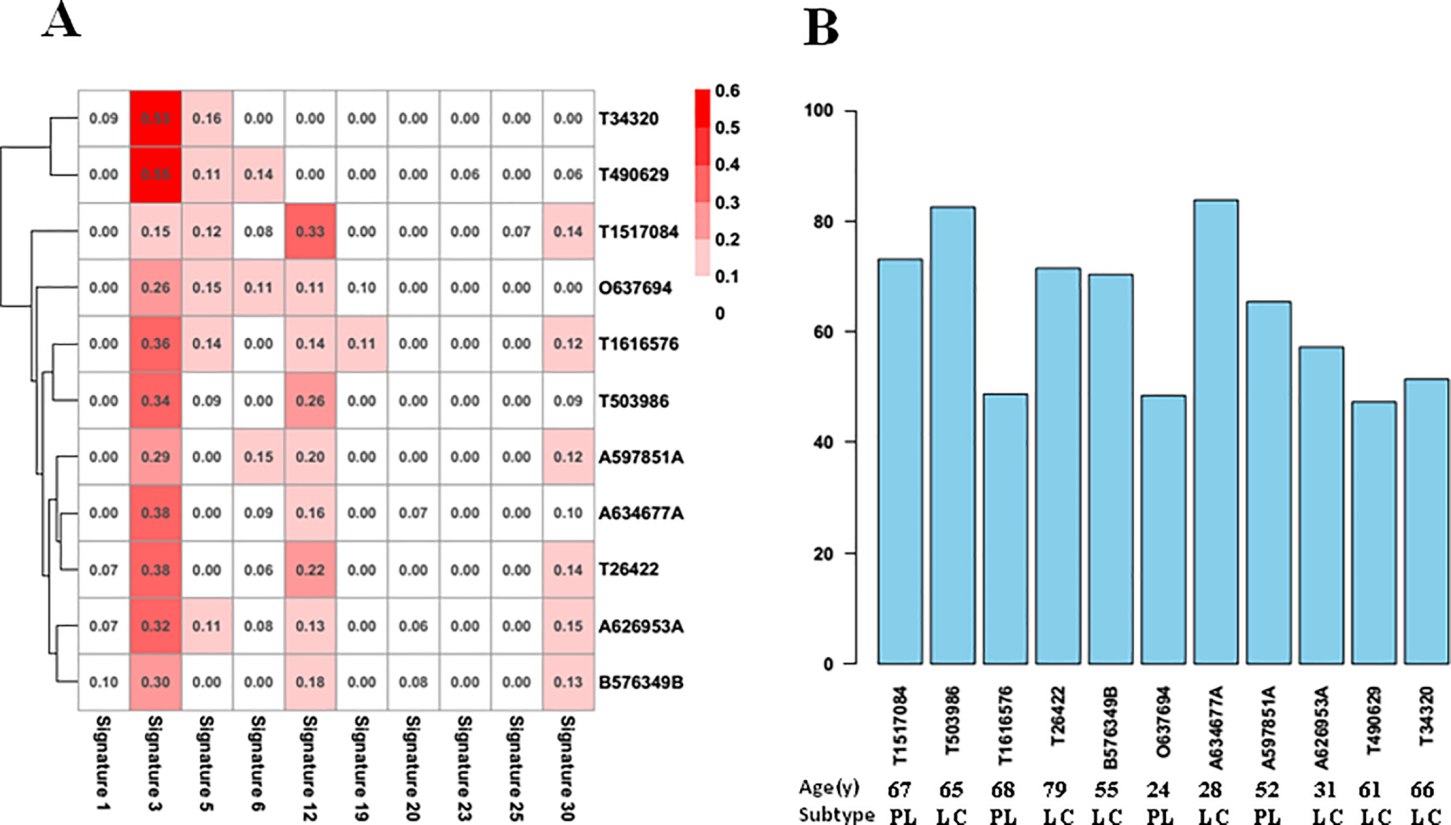
Signatures of mutational processes and intratumor clonal heterogeneity of EBV+ DLBCL cases. The DeconstructSigs method was applied, which uncovered four mutational signatures (Fig 4A), including signatures 3, 5, 12, and 30. Among these signatures, signature 3 was the main type. MATH scores were used to evaluate intratumor clonal heterogeneity (Fig 4B), revealing allele frequency distribution values of between 47 and 86 for the tumors.

To identify the potential contributions of mutational processes within single EBV+ DLBCL genomes, we applied the deconstructSigs method to this mutation set of 11 EBV+ DLBCL tumors. This analysis uncovered four mutational signatures (Fig 4A): signatures 3, 5, 12, and 30 (http://cancer.sanger.ac.uk/cosmic). The main type was signature 3 (Fig 4A), which was characterized by C>G, C>A, and C>T mutations, associated with failure of DNA double-strand break repair by homologous recombination, and strongly related to elevated numbers of large (>3 bp) insertions and deletions with overlapping microhomology at breakpoint junctions. Signatures 5 and 12 were both characterized by T>C, C>T, and C>A mutations. Although both etiologies are unknown, signature 12 has been found in liver cancer samples, while signature 5 has been found in all cancer types and most cancer samples and exhibits a strong transcriptional strand bias for T>C substitutions. Signature 30 was characterized by predominant C>T mutations. Although its etiology is unknown, it has been observed in a small subset of breast cancers samples. Notably, tumors 2 (Patho NO.T 503986) and 7 (Patho NO.A634677) displayed higher values, indicating possible sampling of different subclonal populations or significant differences in tumor heterogeneity between cases.

### Modified MATH method to analyze EBV+ DLBCL heterogeneity

Using the MATH score method [16, 17], the score of mutant-allele fractions among loci will tend to be higher for a heterogeneous tumor than a homogeneous tumor. Fig 4B (right side) presents a bar diagram of the allele frequency distributions in each sample, with values ranging from 0 to 100, which are positively correlated with intratumor heterogeneity. The median allele frequency distribution values for the tumors range from 47 to 86 (Fig 4B), showing considerable variation in MATH scores and indicating that this lymphoma has high heterogeneity. The values for each case may theoretically be influenced by subclonal contributions. Analysis of the MATH scores and the clinical variables of the EBV+ DLBCL cases revealed that the MATH score was strongly associated with Ann Arbor tumor stage. Ann Arbor stage II tumors had low MATH scores, while Ann Arbor stage III–IV tumors had significantly higher MATH scores (*P* = 0.016) (Table 4). More advanced tumors had greater tumor heterogeneity (more tumor subclones), which is consistent with the hypothesis that greater heterogeneity promotes selection of chemotherapy-resistant and more aggressive clones. MATH scores were also significantly correlated with extranodal lesion sites (≥2) (*P* = 0.042) (Table 4), corresponding to the relationship with Ann Arbor stage. On the other hand, MATH scores were not significantly correlated with age, B symptoms, proliferation index, therapy scheme, response to therapy, or status.

**Table 4:** Relationship between clinical variables and MATH scores and OS in 11 EBV+ DLBCL cases.

Variable	Value	(%) No./Total number	Relation to MATH scores	
			Mean ± SD	*P*
**Age at diagnosis**	<50 years old	27% (3/11)	63.67 ± 19.66	0.921
	≥50 years old	73% (8/11)	63.25 ± 13.03	
**Extranodal lesion sites**	<2	64% (7/11)	57.29 ± 10.9	0.042
	≥2	36% (4/11)	74.00 ± 13.37	
**B symptoms**	no	30% (3/10)	56.67 ± 8.02	0.267
	yes	70% (7/10)	68.00 ± 15.24	
**Ann Arbor Stage**	Stage I–II	44% (4/9)	48.75 ± 2.06	0.016
	Stage III–IV	56% (5/9)	72.20 ± 12.26	
**Proliferation index (%)**	<50%	33% (3/9)	61.67 ± 12.37	0.714
	≥50%	67% (6/9)	63.33 ± 13.26	
**Therapy scheme**	rituximab)	67% (6/9)	49.00 ± 0.00	0.286
	Without rituximab	33% (3/9)	67.83 ± 15.32	
**Response to therapy**	CR	44% (4/9)	70.50 ± 16.98	0.286
	PD	56% (5/9)	58.6 ± 11.91	
Status	AWD	70% (7/10)	65.29 ± 14.99	0.517
	DOD	30% (3/10)	56.00 ± 13.23	

Abbreviations: MATH: Mutant-Allele Tumor Heterogeneity; OS, overall survival; EBV+ DLBCL, Epstein-Barr virus-positive diffuse large B-cell lymphoma; CR, complete response; PD, progressive disease; AWD, alive with disease; DOD, died of disease. Values are shown as mean ± SD. *P* values determined by non-parametric Wilcoxon rank-sum test.

### Selected variants in EBV+ DLBCL identified by WES

**Fig 5:**
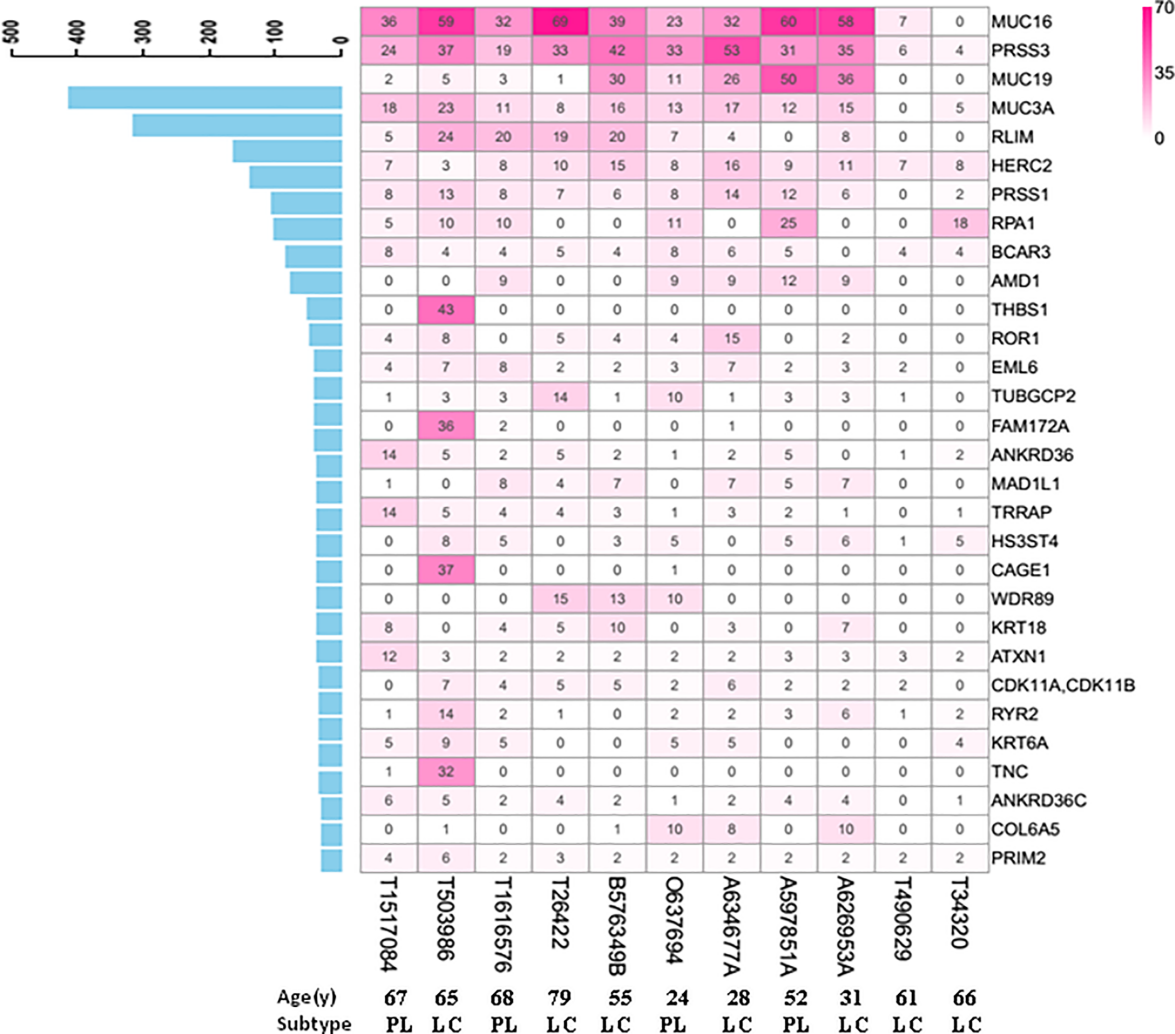
The 30 most commonly mutated genes from selected variants in EBV+ DLBCL identified by whole-exome sequencing. Based on the total number of all mutation loci within one somatic mutated gene in a detected region (exonic region, intronic region, intergenic region, UTR, and other regions), the top 5 most frequently mutated genes were *MUC16*, *PRSS3*, *MUC19*, *MUC3A*, and *RLIM* in decreasing order.

Among the 30 top mutated genes, 14 were mutated at a rate significantly higher than expected by chance in at least two samples. All 11 EBV+ DLBCL cases harbored mutations in at least 1 of these 30 genes (Fig 5). Based on the total number of all mutation loci having one somatic mutated gene in a region analyzed by WES (exonic region, intronic region, intergenic region, UTR, and other regions), we confirmed that the top 10 most frequent mutations were in *MUC16* (10/11 cases), *PRSS3* (11/11 cases), *MUC19* (9/11 cases), *MUC3A* (10/11 cases), *RLIM* (8/11 cases), *HERC2* (11/11 cases), *PRSS1* (10/11 cases), *RPA1* (6/11 cases), *BCAR3* (10/11 cases), and *AMD1* (5/11 cases) (Fig 5).

The gene with the highest number of mutations, *MUC16*, harbored four mutation loci, three of which were located at exon55 (NM_024690: c.G40570A:p.V13524I; c.G40552T:p.V13518L; c.G40588A: p.G13530S), and the remaining loci was at exon 39 (c.G39074A:p.G13025E). All of these mutations were nonsynonymous SNVs. The gene with the second highest number of mutations, *PRSS3*, showed a frameshift deletion/insertion at exon 3, with four mutation loci. The total mutation numbers of these genes were not related to age or pathological subtype (data not shown).

Regarding the frequency of the same mutation locus of one gene of a coding region among all tested samples, *LNP1* mutation (chromosome 3, exon3:c.194_195ins and p.H65del) was the most frequently detected, with all 11 cases showing non-frameshift insertion. The vast majority of *PRSS3* mutations (10/11; 90.9%) were frameshift deletion/insertion, both affecting exon 3 of chromosome 9. Another frequently mutated gene, *MUC3A* (9/11 cases), showed many types of mutations, including nonsynonymous SNV, frameshift insertion, non-frameshift deletion, and non-frameshift insertion, all of which affected exon 2 of chromosome 7. Similar to *MUC3A*, *FADS6* also showed non-frameshift insertion in 9/11 cases. *TRAK1* mutation was found in 8 cases, with all mutations being non-frameshift insertion changes. S4 Table presents the complete list of 57 selected mutated genes.

### Sanger sequencing validation in independent EBV+ DLBCL cases

To verify the accuracy of the top five genetic variants identified from our exome sequencing data (*LNP1*, *PRSS3*, *MUC3A*, *FADS6*, and *TRAK1*), we performed Sanger sequencing (Primer sequences were listed in S6 Table) of 11 our EBV+ DLBCL samples, as well as an additional 16 EBV+ DLBCL samples from another hospital (S7 Table). Results of the two methods were in agreement for over 80% of the assessed variants, confirming the accuracy of our sequencing and bioinformatics methods.

## Discussion

In the present study, we explored the mutational landscape of EBV+ DLBCL with the aim of identifying mutational signatures and tumor heterogeneity. Our main findings were the discoveries of four main signatures matched with the COSMIC database, and high intratumor heterogeneity as shown by MATH scores that were significantly correlated with Ann Arbor stage and ≥2 extranodal lesion sites. Additionally, we identified frequently recurrent mutations of the *LNP1*, *MUC16*, *PRSS3*, and *MUC3A* genes, highlighting previously undetected genetic alteration in the pathogenesis of EBV+ DLBCL.

Among the four main signatures of EBV+ DLBCL matched with COSMIC database, signature 3 was the most representative. Signature 3 was associated with dysfunction of DNA repair by homologous recombination, and strongly related to elevated numbers of large insertions and deletions at breakpoint junctions. A great number of DNA damage response (DDR) and repair proteins mediate and regulate *IG* diversification processes. In a previous study, de Miranda et al. [18] performed targeted sequencing of 73 key DNA repair genes in 29 B-cell lymphoma samples, including 22 DLBCLs. Their WES results revealed more somatic mutations in mismatch repair (MMR)-mutated cases displaying instability of microsatellite markers compared to in microsatellite-stable (MSS) tumors [18]. Additionally, DLBCL carrying the AG/GG genotype of *MLH1* (a component of the DNA mismatch repair system) shows increased risk of death compared to cases harboring the AA genotype, and multivariate analysis adjusted for IPI has identified *MLH1* AG/GG as an independent OS predictor [19].

In our present study, the most common substitution in EBV+ DLBCL was the transition C>T/G>A, which is also the most common mutation in cancer. In most cancer types with kataegis (localized hypermutation), the mutational signatures indicate an association with apolipoprotein B messenger RNA-editing, enzyme-catalytic, polypeptide-like 3 (APOBEC3) enzymes (DNA cytidine deaminases that remove the amino group from a cytosine, converting it to uracil), and show strong enrichment of C-to-T transitions [20]. Such APOBEC3 enzyme activity is reportedly involved in the pathogenesis of B-cell lymphomas, including primary effusion lymphoma [21]. By gene profiling analysis, Yoon et al. [10] identified the host immune response as the key molecular signature in EBV+ DLBCL, while APOBEC3 proteins reportedly act in the innate host response to viral infection [22]. Yoon et al. [10] also showed overexpression of antiviral response genes, chemokines associated with the innate immune response, in EBV+ DLBCL [10]. Interestingly, APOBEC3 cytidine deaminases can edit the genomes of Epstein-Barr herpes virus both *in vitro* and *in vivo*, and function in the innate immune response to viral infections [23]. The molecular signatures of EBV+ DLBCL must still be confirmed in a larger number of cases; however, the signatures identified in our present study indicate the basic features of this rare lymphoma to some degree.

Our analysis revealed that the percentage of mutations in exonic regions ranged from 3.49%–22.68% among the 11 cases, and we identified 57 selected distinct candidate genes that were mutated in EBV+ DLBCL. In contrast, in primary EBV− DLBCL, Zhang et al. [24] found higher proportions of nonsynonymous mutations of each category, and identified 322 recurrently mutated cancer genes. Previously, Yoon et al. [10] performed comparative genomic hybridization (CGH), and reported relatively few genomic alterations in EBV+ DLBCL compared to in EBV− DLBCL. It is possible that in EBV+ DLBCL, both immune escape/immunosenescence and the oncogenic effects of EBV or its related proteins (e.g., LMP1) may reduce the need for the additional genetic alterations than? are frequently found in EBV− DLBCL.

We performed MATH score analysis [16, 17] to evaluate intratumor heterogeneity. WES provides the mutant-allele fraction (MAF) within the total sequenced DNA. Because each genomic locus has a tumor-specific mutation, the MAF value at any genomic locus is influenced both by the presence of subclonal mutations and by CNAs. Our results demonstrated median allele frequency distribution values of between 47 and 86, with high variability of MATH scores among cases, indicating that EBV+ DLBCL harbored high intratumor heterogeneity.

Importantly, heterogeneity reflects the presence of different subclonal populations within the tumor, and likely influences the patient’s clinical course and response to therapy. In esophageal adenocarcinoma, higher intratumor heterogeneity index is strongly correlated with poor responses to neoadjuvant chemotherapy [25]. The MATH scores of our EBV+ DLBCL samples confirmed that higher MATH scores were indicative of increased tumor heterogeneity and were correlated with higher disease stage. This indicated that tumor heterogeneity was an important risk factor for tumor progression, andMATH score might be a useful biomarker for staging EBV+ DLBCL. Moreover, higher MATH scores were significantly correlated with extranodal lesion sites (≥2), which is one factor contributing to a high International Prognosis Index (IPI). This suggests that the MATH score might be useful for identifying patients who will likely show an aggressive clinical course and should receive additional therapy.

To determine whether our study identified any novel mutations, we compared our data with the findings of three previous studies addressing genomic alterations in EBV+ DLBCL via CGH and array CGH [9, 10, 26]. Only two of these three studies showed amplification of chromosome arm 9p [10, 26]. Surprisingly, the mutation genes did not overlap among these different studies, suggesting incomplete mutation discoveries in each study. Differences in methodology, diversity of patient populations, and the small numbers of patients likely contribute to this low overlap. The discrepancy between studies also supports the finding of considerable genetic heterogeneity in EBV+ DLBCL, contributing to the observed patterns of disparate mutations and highlighting the importance of biologically validating these findings.

Some genetic studies of EBV− DLBCL also reveal striking genetic heterogeneity. Zhang et al. [24] constructed Venn diagrams depicting the overlap between genes identified in three studies and their own study. The results showed variation even in the genes that overlapped between different studies. When analysis was limited to 17 genes that were mutated in over 10% of the cases, the overlap between the different studies approached 70%. The overlap is still lower for genes having fewer mutation events [24].

The duration of patient follow-up in our study ranged from 4 to 19 months, and three elderly patients died of EBV+ DLBCL with the LCL subtype. The numbers of SNVs, indels, and SNV items (non-synonymous SNV and synonymous SNV) did not differ between age groups or subtype groups, indicating that the genetic spectrum of EBV+ DLBCL was unrelated to age, histopathological subtype, and prognosis. In total, we identified 57 selected distinct candidate genes that showed recurrent somatic mutations in this disease. However, our mutations numbers were likely overestimated due to the lack of corresponding germline DNA for WES.

In recent years, elucidation of the molecular mechanisms responsible for EBV− DLBCL has enabled the identification of multiple mutational hotspots—including *MYD88*, *CD79A*, and *CARD11*—that promote B-cell receptor (BCR) and/or Toll-like receptor (TLR)-mediated activation of NF-kappa B signaling. However, Gebauer et al. [9] found that EBV+ DLBCL exhibited few mutations affecting *CD79B* and *CARD11*, and no mutations in *MYD88*. This suggested that EBV-mediated activation of NF-kB may occur as an alternative to pathologically enforced B-cell receptor signaling in this rare lymphoma.

In our present study, we did not explore the signal pathway for this lymphoma due to a lack of germline DNA, but we detected commonly mutated genes. Genes that showed high mutation numbers and frequencies included serine protease 3 (*PRSS3*), *MUC3A*, and *MUC16*. *PRSS3* is an isoform of trypsinogen, and plays important roles in the development of many malignancies [27, 28]. https://www.ncbi.nlm.nih.gov/pubmed/?term=Fadlelmola%20FM%5BAuthor%5D&cauthor=true&cauthor_uid=18179710Fadlelmola et al. [29] investigated HL-derived cell lines and ALCL cell lines, and identified disease-associated gene copy number gains and losses, and confirmed the amplification of all three isoforms of the trypsin gene (*PRSS1/PRSS2/PRSS3*). This finding raises interesting possibilities regarding the role of signaling pathways triggered by membrane-associated serine proteases in HL and aggressive non-Hodgkin's lymphoma (NHL).

*MUC3A* is a membrane-associated mucin that is involved in cancer pathogenesis and progression [30]. Its expression is an adverse independent prognostic factor for overall survival (OS) and recurrence-free survival (RFS) in localized clear-cell renal cell carcinoma [31]. Until now, no available data have demonstrated that *MUC3A* is related to lymphoma. Further studies are needed to explore the functions of this gene in EBV+ DLBCL.

*MUC16*, also called carbohydrate antigen 125 (*CA125*, encoded by *MUC16*), is reportedly elevated in the serum of patients with NHL, including DLBCL [32, 33], mucosa-associated lymphoid tissue lymphoma [34], follicular lymphoma [35]. *MUC16* is also a prognostic factor in DLBCL [36]. In our present study, *CA125* harbored the highest total number of all mutation loci, and all mutations were of the non-synonymous SNV type. This is the first report showing *CA125* mutation in B-cell lymphoma.

The gene mutated at the highest frequency in all 11 cases was *LNP1*, which is a regulator of cortical endoplasmic reticulum [37]. In EBV+ DLBCL, *LNP1* harbored non-frameshift insertion mutations. Few studies have examined *LNP1*. One study shows that NUP98/11p15 translocations affect the CD34+/CD133+ hematopoietic precursor through one of its partners, *LNP1*, in myeloid and T lymphoid leukemias [38]. *LNP1—*together with other highly mutated genes, such as *FADS6* and *TRAK1—*was firstly found in this lymphoma, and was verified by Sanger sequencing. The functions of these genes in this lymphoma remain to be explored. Overall, our findings suggest new therapeutic possibilities in EBV+ DLBCL, that should be further explored in clinical trials, in conjunction with approaches to assay for these mutations.

In summary, our present whole-exome study of EBV+ DLBCL revealed several genes that may contribute to lymphomagenesis. We further demonstrated the intratumoral heterogeneity and subclonal architecture of EBV+ DLBCL, which may be of relevance in the clinical evolution of these tumors. Notably, the differential distribution of these mutations in EBV+ DLBCL illustrates the relationship between genomic alterations and tumor heterogeneity.

## Supporting information

S1 File English version of verification of medical ethics committee. (PDF)

S1 Table Whole-exome sequencing performance data. (DOCX)

S2 Table All variants shown by whole-exome sequencing (WES) in 11 EBV+ DLBCL cases. (DOCX)

S3 Table Somatic mutations identified by whole-exome sequencing (WES) in 11 EBV+ DLBCL cases. (DOCX)

S4 Table Summary of the total number of single-nucleotide variations (SNVs) andIndels detected by whole-exome sequencing (WES) in 11 EBV+ DLBCL cases. (DOCX)

S5 Table Comparisons of single-nucleotide variations (SNV) and Indel numbers in 11 EBV+ DLBCL according to age and pathological subtype. (DOCX)

S6 Table Validation of the most recurrent genes in 11 EBV+ DLBCL patients analyzed by whole-exome sequencing. (DOC)

S7 Table Clinicobiological features of 16 additional EBV+ DLBCL patients analyzed by Sanger sequencing. (DOCX)
