# Supplementary material for: Genetic heterogeneity and mutational signature in Chinese Epstein-Barr virus-positive diffuse large B-cell lymphoma
Source: PLoS One. 2018 Aug 14;13(8):e0201546. doi: 10.1371/journal.pone.0201546 (PMC6091946; doi:10.1371/journal.pone.0201546)
Supplement: S1 File — (PDF) [file pone.0201546.s001.pdf]

Sun Yat-sen University Foshan hospital
The first people's hospital of Foshan

The opinion of medical ethics committee

The Chinese National Nature Sciences Foundation: The screening for pathogenic genes and clonal heterogeneity in EB virus positive diffuse large B cell lymphoma of the elderly (NO.81302035).

Dr. Fang Liu successfully obtained above research fund. After examination of medical ethics committee of Sun Yat-sen University Foshan hospital, also named as The first people's hospital of Foshan (Number: L[2014] NO.2), it's research methods and contents were coincidence with the rules and regulations of medical ethics committee, so it is permitted to explore the research and publish related papers.

The medical ethics committee of Sun yat-sen University Foshan hospital

2014.2.4

佛山市第一人民医院 医学伦理委员会
