# Supplementary material for: Genetic heterogeneity and mutational signature in Chinese Epstein-Barr virus-positive diffuse large B-cell lymphoma
Source: PLoS One. 2018 Aug 14;13(8):e0201546. doi: 10.1371/journal.pone.0201546 (PMC6091946; doi:10.1371/journal.pone.0201546)
Supplement: S1 Table — (DOCX) [file pone.0201546.s002.docx]

| **S1 Table WES performance data** | | | | | | | | |
| --- | --- | --- | --- | --- | --- | --- | --- | --- |
| **Sample ID** | **Total reads(%)** | **Mapped reads(%)** | **Total data (Mb)** | **Effective sequences on target (Mb)** | **% of bases covered per patient** | **Average sequencing**  **depth on target** | **Coverage of**  **Target region** | **Fraction of target covered with >10x** |
| **EBV+DLBCL1 (T1517084)** | **81017032 (100%)** | **80961286 (99.93%)** | **12152.56** | **8026.49** | **66.0** | **159** | **99.79%** | **99.05%** |
| **EBV+DLBCL2**  **(T503986)** | **89017440 (100%)** | **88628711 (99.56%)** | **13352.62** | **7673.77** | **57.5** | **152** | **99.89%** | **99.41%** |
| **EBV+DLBCL3**  **(T1616576)** | **80050426 (100%)** | **79989339 (99.92%)** | **12007.56** | **8016.53** | **66.8** | **159** | **99.79%** | **99.46%** |
| **EBV+DLBCL4**  **(T26422)** | **81357896 (100%)** | **81303852 (99.93%)** | **12203.68** | **8327.34** | **68.2** | **165** | **99.9%** | **99.53%** |
| **EBV+DLBCL5**  **(B576349B)** | **74030074 (100%)** | **73964642 (99.91%)** | **11104.51** | **8558.08** | **77.1** | **142** | **99.53%** | **97.26%** |
| **EBV+DLBCL6**  **(O637694)** | **71254700 (100%)** | **71173664 (99.89%)** | **10688.21** | **8230.23** | **77.0** | **136** | **99.46%** | **97.42%** |
| **EBV+DLBCL7**  **(A634677A)** | **72561404 (100%)** | **72476590 (99.88%)** | **10884.21** | **8156.23** | **74.9** | **135** | **99.67%** | **97.68%** |
| **EBV+DLBCL8**  **(A597851A)** | **65637122 (100%)** | **65575194 (99.91%)** | **9845.57** | **7652.12** | **77.7** | **127** | **99.53%** | **96.36%** |
| **EBV+DLBCL9**  **(A626953A)** | **58532612 (100%)** | **58480286 (99.91%)** | **8779.89** | **6695.96** | **76.3** | **111** | **99.21%** | **95.12%** |
| **EBV+DLBCL10**  **(T490629)** | **67116188 (100%)** | **65112608 (97.01%)** | **10067.43** | **5744.83** | **57.1** | **114** | **99.87%** | **98.9%** |
| **EBV+DLBCL11**  **(T34320)** | **85902612 (100%)** | **84001473 (97.79%)** | **12885.39** | **7746.98** | **60.1** | **154** | **99.83%** | **98.9%** |

**Abbreviation**:: EBV+DLBCL，EB virus positive diffuse large B cell lymphoma; WES,whole exome sequence
