# Supplementary material for: Genetic heterogeneity and mutational signature in Chinese Epstein-Barr virus-positive diffuse large B-cell lymphoma
Source: PLoS One. 2018 Aug 14;13(8):e0201546. doi: 10.1371/journal.pone.0201546 (PMC6091946; doi:10.1371/journal.pone.0201546)
Supplement: S2 Table — (DOCX) [file pone.0201546.s003.docx]

| **S2 Table all of variants shown by WES in 11 EBV+DLBCL** | | | | | | |
| --- | --- | --- | --- | --- | --- | --- |
| Sample ID | **Exonic** | **Intronic** | **Intergenic** | **Other** | **Total** | **% of mutation**  **amount in exonic regions** |
| **EBV+DLBCL1**  **(T1517084)** | **350** | **5866** | **2700** | **1127** | **10043** | **3.49** |
| **EBV+DLBCL2**  **(T503986)** | **356** | **4741** | **1707** | **1293** | **8097** | **4.4** |
| **EBV+DLBCL3**  **(T1616576)** | **219** | **2276** | **1139** | **721** | **4355** | **5.03** |
| **EBV+DLBCL4**  **(T26422)** | **212** | **2215** | **1112** | **663** | **4202** | **5.05** |
| **EBV+DLBCL5**  **(B576349B)** | **314** | **1825** | **1194** | **660** | **3993** | **7.86** |
| **EBV+DLBCL6**  **(O637694)** | **382** | **1694** | **1087** | **645** | **3808** | **10.03** |
| **EBV+DLBCL7**  **(A634677A)** | **200** | **1766** | **1191** | **619** | **3776** | **5.3** |
| **EBV+DLBCL8**  **(A597851A)** | **270** | **1480** | **1270** | **579** | **3599** | **7.5** |
| **EBV+DLBCL9**  **(A626953A)** | **378** | **1450** | **1097** | **569** | **3494** | **10.82** |
| **EBV+DLBCL10**  **(T490629)** | **410** | **788** | **363** | **247** | **1808** | **22.68** |
| **EBV+DLBCL11**  **(T34320)** | **235** | **661** | **249** | **207** | **1352** | **17.38** |

**Abbreviation**: EBV+DLBCL , Epstein-Barr virus positive diffuse large B cell lymphoma.WES，Whole exome sequence
