# Supplementary material for: Genetic heterogeneity and mutational signature in Chinese Epstein-Barr virus-positive diffuse large B-cell lymphoma
Source: PLoS One. 2018 Aug 14;13(8):e0201546. doi: 10.1371/journal.pone.0201546 (PMC6091946; doi:10.1371/journal.pone.0201546)
Supplement: S3 Table — (DOCX) [file pone.0201546.s004.docx]

**S3 Table Somatic mutations indentified by WES in 11 EBV+DLBCLs**

| Gene | Chr | Position | Nucleotide change | Codon changes :AA changes | Founction class | Number of case |
| --- | --- | --- | --- | --- | --- | --- |
| LNP1 | **chr3** | **100170600** | **A/ ATCCTA** | **c.194_195insTCCTAGAAGGCATTCTCATGAGGACCAGGAATTCCGATGCCGATCGTCTGACCGTCT**  **p.H65delinsHPRRHSHEDQEFRCRSSDRL** | **nonframeshift_insertion** | **11** |
| PRSS3 | **chr9** | **33797930** | **GAC/G** | **c.284_285del:p.D95fs; c.305_306del:p.D102fs; c.476_477del:p.D159fs,**  **c.347_348del:p.D116fs** | **frameshift_deletion** | **10** |
|  |  | **33797928** | **G/ GCC** | **c.281_282insCC:p.R94fs; c.302_303insCC:p.R101fs,**  **c.473_474insCC:p.R158fs; c.344_345insCC:p.R115fs** | **frameshift_insertion** | **10** |
| MUC3A | **Chr7** | **100550515** | **T/A** | **c.T1096A:p.S366T** | **nonsynonymous_SNV** | **9** |
|  |  | **100550490** | **G/A** | **c.G1071A:p.M357I** | **nonsynonymous_SNV** | **9** |
|  |  | **100550469** | **A/C** | **c.A1050C:p.K350N** | **nonsynonymous_SNV** | **5** |
|  |  | **100550472** | **C/CAG** | **c.1053_1054insAG:p.I351fs** | **frameshift_insertion** | **5** |
|  |  | **100550510** | **A/C** | **c.A1091C:p.E364A** | **nonsynonymous_SNV** | **6** |
|  |  | **100550571** | **G** | **c.G1152A:p.M384I** | **nonsynonymous_SNV** | **3** |
|  |  | **100550638** | **ATCTACT** | **c.1220_1225del:p.407_409del** | **nonframeshift_deletion** | **2** |
|  |  | **100550632** | **T** | **c.1213_1214insCG:p.S405fs** | **frameshift_insertion** | **2** |
|  |  | **100550630** | **C** | **c.1212dupA:p.S404fs** | **frameshift_insertion** | **2** |
|  |  | **100550486** | **G** | **c.G1067A:p.S356N** | **nonsynonymous_SNV** | **2** |
|  |  | **100550625** | **C** | **c.1206_1207insACT:p.F402delinsFT** | **nonframeshift_insertion** | **2** |
| FADS6 | **Chr17** | **72889676** | **G/GGGCTCCGTAGGTTCCATGGGCTCCGTAGGTTCCATGGGCTCCGTAGGTTCCATC** | **c.17_18insGATGGAACCTACGGAGCCCATGGAACCTACGGAGCCCATGGAACCTACGGAGCC: p.P6delinsPMEPTEPMEPTEPMEPTEP** | **nonframeshift_insertion** | **9** |
| TRAK1 | **chr3** | **42251577** | **C/ CGGA; CGGAGGA** | **c.1841_1842insGGAGGA:p.T614delinsTEE,**  **c.1889_1890insGGAGGA:p.T630delinsTEE,**  **c.2063_2064insGGAGGA:p.T688delinsTEE** | **nonframeshift_insertion** | **8** |
| PABPC3 | **Chr13** | **25670801** | **T/TG** | **c.465_466insG:p.I155fs** | **frameshift_insertion** | **6** |
| PCLO | **chr7** | **82784833** | **T /TGAGCTGGAGGCTTAGC**  **AGGACCAAGAG** | **c.1123_1124insCTCTTGGTCCTGCTAAGCCTCCAGCTC:**  **p.Q375delinsPLGPAKPPAQ** | **nonframeshift_insertion** | **6** |
| MUC16 | **chr19** | **9000187** | **C/T** | **c.G40570A:p.V13524I** | **nonsynonymous_SNV** | **6** |
|  |  | **9000169** | **C** | **c.G40588A:p.G13530S** | **nonsynonymous_SNV** | **4** |
|  |  | **9009652** | **C** | **c.G39074A:p.G13025E** | **nonsynonymous_SNV** | **3** |
| KDM6B | **Chr17** | **7750177** | **TACCACC/ ,TACC;T,TACCACCACC; T,TACCACCACCACCACCACC** | **c.756_758del:p.252_253del** | **nonframeshift_deletion** | **6** |
| ATN1 | **chr12** | **7045891** | **A/ ACAG,ACAGCAG; ACAGCAGCAGCAG; ,ACAGCAGCAGCAGCAGCAGCAG** | **c.1461_1462insCAGCAG:p.Q487delinsQQQ;** | **nonframeshift_insertion** | **5** |
| KRT18 | **chr12** | **53343231** | **G/C** | **c.G274C:p.A92P;**  **c.G274C:p.A92P** | **nonsynonymous_SNV** | **5** |
|  |  | **53343148** | **C** | **c.C191G:p.A64G；**  **c.C191G:p.A64G** |  | **4** |
|  |  | **53343225** | **C** | **c.C268T:p.R90C；**  **c.C268T:p.R90C** |  | **4** |
|  |  | **53343099** | **G** | **c.G142A:p.V48M；**  **c.G142A:p.V48M** |  | **4** |
|  |  | **53343265** | **C** | **c.C308A:p.T103N；**  **c.C308A:p.T103N** |  | **3** |
|  |  | **53343132** | **G** | **c.G175T:p.G59W；c.G175T:p.G59W** |  | **3** |
|  |  | **53343131** | **G** | **c.174_175insA:p.M58fs**  **c.174_175insA:p.M58fs** | **frameshift_insertion** | **3** |
|  |  | **53343257** | **C** | **c.C300G:p.S100R,KRT18；**  **c.C300G:p.S100R** | **nonsynonymous_SNV** | **3** |
| BCR | **chr22** | **23653975** | **T/TCCGG** | **c.3142_3143insCCGG:p.S1048fs;**  **c.3274_3275insCCGG:p.S1092fs** | **frameshift_insertion** | **5** |
|  |  | **23654017** | **G** | **c.G3184A:p.D1062N；**  **c.G3316A:p.D1106N** | **nonsynonymous_SNV** | **3** |
| USF3 | **Chr3** | **113376110** | **TTGCTGC /T,TTGCTGC** | **c.4413_4415del:p.1471_1472del** | **nonframeshift_deletion** | **5** |
| RPL14 | **chr3** | **40503520** | **ACTGCTGCTGCTG** | **c.457_458insCTGCTGCTG:p.A153delinsAAAA；**  **c.457_458insCTGCTGCTG:p.A153delinsAAAA** | **nonframeshift_insertion** | **4** |
| ZNF77 | **chr19** | **2939267** | **CACCACCCTTACCCAAGGAGGCA** | **c.120_130del:p.L40fs** | **frameshift_deletion** | **4** |
| MEF2A | **chr15** | **100252709** | **CCAG** | **c.1026_1027insCAGCAG:p.Q342delinsQQQ,MEF2A: c.1230_1231insCAGCAG:p.Q410delinsQQQ,MEF2A:**  **c.1050_1051insCAGCAG:p.Q350delinsQQQ,MEF2A:**  **c.1236_1237insCAGCAG:p.Q412delinsQQQ,MEF2A:**  **c.1230_1231insCAGCAG:p.Q410delinsQQQ** | **nonframeshift_insertion** | **4** |
| MUC4 | **chr3** | **195513413** | **G** | **c.5037_5038insTCTCTTCCTGTCACCAGCACTTCCTCAGCATCCACCGGTCACGCCACCCCTCTTCCTGTCACCGACAATTCCTCAGTATCCACAGGTCACGCCACC:p.P1680delinsSLPVTSTSSASTGHATPLPVTDNSSVSTGHATP** | **nonframeshift_insertion** | **4** |
|  |  | **195507092** | **T** | **c.A11359T:p.T3787S** | **nonsynonymous_SNV** | **3** |
|  |  | **195510749** | **C** | **c.7701_7702insTCAGTATCCACAGGTCATGCCACCCCTCTTCATGTCACCGACACTTCC:p.A2568delinsSVSTGHATPLHVTDTSA** | **nonframeshift_insertion** | **2** |
|  |  | **195507109** | **T** | **c.A11342T:p.D3781V** | **nonsynonymous_SNV** | **2** |
| SLC9B1 | **chr4** | **103826757** | **T** | **c.A1246G:p.T416A,SLC9B1:**  **c.A1246G:p.T416A** | **nonsynonymous_SNV** | **4** |
| USP17L17 | **chr4** | **9245671** | **T** | **c.T67C:p.S23P** | **nonsynonymous_SNV** | **4** |
| ATXN3 | **chr14** | **92537354** | **C** | **c.67_68insCAGCAGCAGCAGCAGCAGCAGCAGCAGCAGCAGCA:p.G23fs; c.232_233insCAGCAGCAGCAGCAGCAGCAGCAGCAGCAGCAGCA:p.G78fs**  **c.112_113insCAGCAGCAGCAGCAGCAGCAGCAGCAGCAGCAGCA:p.G38fs**  **c.277_278insCAGCAGCAGCAGCAGCAGCAGCAGCAGCAGCAGCA:p.G93fs**  **c.430_431insCAGCAGCAGCAGCAGCAGCAGCAGCAGCAGCAGCA:p.G144fs**  **c.552_553insCAGCAGCAGCAGCAGCAGCAGCAGCAGCAGCAGCA:p.G185fs**  **c.378_379insCAGCAGCAGCAGCAGCAGCAGCAGCAGCAGCAGCA:p.G127fs**  **c.762_763insCAGCAGCAGCAGCAGCAGCAGCAGCAGCAGCAGCA:p.G255fs**  **c.705_706insCAGCAGCAGCAGCAGCAGCAGCAGCAGCAGCAGCA:p.G236fs**  **c.870_871insCAGCAGCAGCAGCAGCAGCAGCAGCAGCAGCAGCA:p.G291fs**  **c.750_751insCAGCAGCAGCAGCAGCAGCAGCAGCAGCAGCAGCA:p.G251fs**  **c.915_916insCAGCAGCAGCAGCAGCAGCAGCAGCAGCAGCAGCA:p.G306fs** | **frameshift_insertion** | **4** |
| ATXN1 | **chr6** | **16327915** | **ATGC** | **c.624_626del:p.208_209del,ATXN1:NM_000332:exon8:c.624_626del:p.208_209del** | **nonframeshift_deletion** | **4** |
| PABPC3 | **chr13** | **25671159** | **G** | **c.G823T:p.E275X** | **stopgain** | **4** |
|  |  | **25671141** | **A** | **c.A805T:p.K269X** | **stopgain** | **3** |
|  |  | **25671163** | **TTAAGC** | **c.828_832del:p.L276fs** | **frameshift_deletion** | **3** |
| 333GOLGA8K | **chr15** | **32685308** | **G** | **c.C1652T:p.P551L** | **nonsynonymous_SNV** | **3** |
| WDR89 | **chr14** | **64066352** | **T** | **c.A309T:p.R103S; c.A309T:p.R103S; c.A309T:p.R103S** | **nonsynonymous_SNV** | **3** |
|  |  | **64066402** | **A** | **c.T259C:p.S87P; c.T259C:p.S87P; c.T259C:p.S87P** |  | **3** |
|  |  | **64066395** | **C** | **c.G266A:p.C89Y; c.G266A:p.C89Y; c.G266A:p.C89Y** |  | **3** |
|  |  | **64066398** | **G** | **c.C263G:p.A88G; c.C263G:p.A88G; c.C263G:p.A88G** |  | **3** |
|  |  | **64066363** | **G** | **exon3:c.C298T:p.R100X； exon4:c.C298T:p.R100X** | **stopgain** | **3** |
|  |  | **64066367** | **A** | **exon3:c.T294A:p.D98E; exon4:c.T294A:p.D98E** | **nonsynonymous_SNV** | **3** |
|  |  | **64066303** | **T** | **exon3:c.A358C:p.S120R; exon4:c.A358C:p.S120R** | **nonsynonymous_SNV** | **2** |
|  |  | **64066302** | **C** | **exon3:c.G359A:p.S120N; exon4:c.G359A:p.S120N** | **nonsynonymous_SNV** | **2** |
|  |  | **64066326** | **C** | **exon3:c.G335A:p.G112D; exon4:c.G335A:p.G112D** | **nonsynonymous_SNV** | **2** |
|  |  | **1018312** | **G** | **c.C4489T:p.H1497Y** | **nonsynonymous_SNV** | **3** |
| MUC6 | **chr11** | **1018290** | **G** | **c.C4511G:p.P1504R;** | **nonsynonymous_SNV** | **3** |
|  |  | **1018321** | **C** | **c.G4480A:p.G1494R** | **nonsynonymous_SNV** | **2** |
|  |  | **1018312** | **G** | **c.C4489T:p.H1497Y** | **nonsynonymous_SNV** | **3** |
| TAS2R46 | **chr12** | **11214001** | **C** | **c.G893C:p.R298T** | **nonsynonymous_SNV** | **3** |
| RSPH6A | **chr19** | **46299138** | **T** | **c.2142_2143insGAGGAGGAGGGCGAGGAG:p.T715delinsEEEGEET** | **nonframeshift_insertion** | **3** |
| ERICH6 | **Chr3** | **150421527** | **C** | **c.158_159insAGAGGTGGAGGAGGAGGAGGA:p.E53delinsEEVEEEEE** | **nonframeshift_insertion** | **3** |
| GXYLT1 | **chr12** | **42538367** | **C** | **c.G82A:p.V28M; c.G82A:p.V28M** | **nonsynonymous_SNV** | **3** |
|  |  | **42538366** | **A** | **c.T83A:p.V28E,GXYLT1;c.T83A:p.V28E** | **nonsynonymous_SNV** | **3** |
|  |  | **42538349** | **T** | **c.A100G:p.T34A,GXYLT1; c.A100G:p.T34A** | **nonsynonymous_SNV** | **3** |
|  |  | **42538340** | **C** | **c.G109T:p.G37C,GXYLT1;c.G109T:p.G37C** | **nonsynonymous_SNV** | **3** |
|  |  | **42538352** | **C** | **c.G97T:p.G33X,GXYLT1;c.G97T:p.G33X** | **stopgain** | **3** |
| KRT10 | **chr17** | **38975103** | **A** | **c.1683_1684insAGCTCCGGCGGCGGATACGGCGGCGGCAGCAGCTCCGGCGGCGGATACGGCGGCGGCAGC:p.S562delinsSSGGGYGGGSSSGGGYGGGSS** | **nonframeshift_insertion** | **3** |
| HTT | **chr4** | **3076654** | **G** | **c.102_103insCAA:p.Q34delinsQQ** | **nonframeshift_insertion** | **3** |
| ASPN | **chr9** | **95237024** | **CTCATCA** | **c.150_152del:p.50_51del,ASPN**  **c.150_152del:p.50_51del** | **nonframeshift_deletion** | **3** |
| ZNF384 | **chr12** | **6777069** | **TTGCTGC** | **c.1191_1193del:p.397_398del,**  **c.1356_1358del:p.452_453del,**  **c.1539_1541del:p.513_514del** | **nonframeshift_deletion** | **3** |
| PPP2R2B | **chr5** | **146258290** | **A** | **c.57_58insAGCAGCAGCAGCAGCAGCAGC:p.C20delinsSSSSSSSC** | **nonframeshift_insertion** | **3** |
| ALMS1 | **chr2** | **73613031** | **TGGAGGA** | **c.41_42insGGAGGA:p.E14delinsEEE** | **nonframeshift_insertion** | **3** |
| KRTAP5-7 | **chr11** | **71238675** | **C** | **c.329_330insCTGCTGCCAGTCCAGCTGCTGTAAGCCCTGCTGCTGCCAGTCCAGCTGCTGTAAGCCCTG:p.S110delinsSCCQSSCCKPCCCQSSCCKPC** | **nonframeshift_insertion** | **3** |
| FDFT1 | **chr8** | **11666218** | **GTCCCAC** | **c.198_199insTCCCACTCCCAC:p.H66delinsHSHSH** | **nonframeshift_insertion** | **2** |
| SENP7 | **chr3** | **101136452** | **A** | **c.T368G:p.L123R, c.T467G:p.L156R** | **nonsynonymous_SNV** | **2** |
| ANKRD36 | **chr2** | **97851073** | **GC** | **c.1961delC:p.A654fs** | **frameshift_deletion** | **2** |
| FLG | **chr1** | **152278775** | **T** | **c.A8587T:p.T2863S** | **nonsynonymous_SNV** | **2** |
|  |  | **152278770** | **ATG** | **c.8590_8591del:p.H2864fs** | **frameshift_deletion** | **2** |
|  |  | **152278768** | **G** | **c.8593_8594insGG:p.A2865fs** | **frameshift_insertion** | **2** |
| LOC283710 | **chr15** | **31521505** | **CGG** | **c.75delC:p.P25fs** | **frameshift_deletion** | **2** |
| RP1L1 | **chr8** | **10467589** | **T** | **c.4018_4019insGGACTAAAGTAATAGAAGGGCTGCAAGAAGAGAGGGTGCAGTTAGAGG:p.E1340delinsGTKVIEGLQEERVQLEE** | **nonframeshift_insertion** | **2** |
| IGLL5 | **chr22** | **23230361** | **T** | **c.T128C:p.V43A** | **nonsynonymous_SNV** | **2** |
| ANKLE1 | **chr19** | **17397456** | **GGTGT** | **c.1892_1893insGT:p.V631fs** | **frameshift_insertion** | **2** |
| MUC17 | **chr7** | **100680296** | **A** | **c.5600dupC:p.T1867fs** | **frameshift_insertion** | **2** |
|  |  | **100680293** | **AG** | **c.5597delG:p.S1866fs** | **frameshift_deletion** | **2** |
| MYBBP1A | **chr17** | **4443006** | **T** | **c.A3691T:p.K1231X** | **stopgain** | **2** |
| KLF2 | **chr19** | **16436170** | **C** | **c.C219G:p.F73L** | **nonsynonymous_SNV** | **2** |
| ZNF257 | **chr19** | **22270899** | **G** | **exon2:c.G119T:p.G40V; c.G347T:p.G116V; c.G251T:p.G84V; c.G119T:p.G40V** | **nonsynonymous_SNV** | **2** |
| VCL | **chr10** | **75758117** | **T** | **c.T152G:p.V51G;** | **nonsynonymous_SNV** | **2** |
| ANKRD36 | **chr2** | **97851073** | **GC** | **c.1961delC:p.A654fs** | **frameshift_deletion** | **2** |
|  |  | **97851078** | **A** | **c.1965_1966insG:p.S655fs** | **frameshift_insertion** | **2** |
| CAPRIN1 | **chr11** | **34074047** | **A** | **c.A80C:p.E27A** | **nonsynonymous_SNV** | **2** |
| GALNT14 | **chr2** | **31165076** | **C** | **c.G922C:p.E308Q; c.G937C:p.E313Q;c.G862C:p.E288Q** | **nonsynonymous_SNV** | **2** |
| FAM120B | **chr6** | **170627695** | **A** | **c.1253_1254insCCCTGAACCCAGGCAAGAAGTTCCCATGTGTACAGG:p.D418delinsDPEPRQEVPMCTG,FAM120B:**  **c.1286_1287insCCCTGAACCCAGGCAAGAAGTTCCCATGTGTACAGG:p.D429delinsDPEPRQEVPMCTG,FAM120B:**  **c.1217_1218insCCCTGAACCCAGGCAAGAAGTTCCCATGTGTACAGG:p.D406delinsDPEPRQEVPMCTG** | **nonframeshift_insertion** | **2** |
| OR2T29 | **chr1** | **248722788** | **G** | **c.C5A:p.A2D** | **nonsynonymous_SNV** | **2** |
| FAM157B | **chr9** | **141107536** | **GGCAGCAGCAGCA** | **exon2:c.230_231insGCAGCA:p.Q77delinsQQQ** | **nonframeshift_insertion** | **2** |
| ATXN2 | **Chr12** | **112036753** | **GGCTGCTGCTGCT** | **c.554_556del:p.185_186del** | **nonframeshift_deletion** | **2** |
| TMEM247 | **Chr2** | **46707808** | **C** | **c.382_383insAGCGGCAGCACGAGGTGGTGATGGAGCAGCTGCAGCGGG:p.Q128delinsQRQHEVVMEQLQRE** | **nonframeshift_insertion** | **2** |

**Abbreviations**: AA, amino acid; WES, whole-exome sequence; EBV+DLBCL，E pstein-Barr virus positive diffuse large B cell lymphoma
