# Supplementary material for: Genetic heterogeneity and mutational signature in Chinese Epstein-Barr virus-positive diffuse large B-cell lymphoma
Source: PLoS One. 2018 Aug 14;13(8):e0201546. doi: 10.1371/journal.pone.0201546 (PMC6091946; doi:10.1371/journal.pone.0201546)
Supplement: S4 Table — (DOCX) [file pone.0201546.s005.docx]

**S4 Table Summary of the total number of SNVs and Indels detected by WES in 11 EBV+DLBCL cases**

| **Sample ID** | **Age** | **Subtype** | **SNV** | **Indel** | **Total** | **SNV items** | | |
| --- | --- | --- | --- | --- | --- | --- | --- | --- |
|  |  |  |  |  |  | **Nonsynonymous SNV** | **Synonymous SNV** | **Other** |
| EBV+DLBCL1 (T1517084) | **67** | **PL** | **7379** | **2671** | **10050** | **188** | **76** | **86** |
| EBV+DLBCL2  (T503986) | **65** | **LCL** | **3594** | **4517** | **8111** | **141** | **149** | **66** |
| EBV+DLBCL3 (T1616576) | **68** | **PL** | **2116** | **2244** | **4360** | **108** | **60** | **51** |
| EBV+DLBCL 4 (T26422) | **79** | **LCL** | **2143** | **2068** | **4211** | **126** | **52** | **34** |
| EBV+DLBCL 5 (B576349B) | **55** | **LCL** | **2594** | **1403** | **3997** | **166** | **81** | **67** |
| EBV+DLBCL 6 (O637694) | **24** | **PL** | **2529** | **1285** | **3814** | **230** | **86** | **66** |
| EBV+DLBCL7 (A634677A) | **28** | **LCL** | **2310** | **1466** | **3776** | **105** | **56** | **39** |
| EBV+DLBCL 8 (A597851A) | **52** | **PL** | **2488** | **1120** | **3608** | **152** | **57** | **61** |
| EBV+DLBCL 9 (A626953A) | **31** | **LCL** | **2415** | **1087** | **3502** | **224** | **97** | **57** |
| EBV+DLBCL 10 (T490629) | **61** | **LCL** | **1522** | **288** | **1810** | **252** | **109** | **49** |
| EBV+DLBCL11 (T34320) | **66** | **LCL** | **1132** | **220** | **1352** | **151** | **63** | **21** |

**Abbreviation**: EBV+DLBCL, Epstein-Barr virus positive diffuse large B cell lymphoma; PL, polymorphous lymphoma; LCL, large cell lymphoma ; SNV, Single nucleotied variant; Indel, Insertion/Deletion;
