# Supplementary material for: Genetic heterogeneity and mutational signature in Chinese Epstein-Barr virus-positive diffuse large B-cell lymphoma
Source: PLoS One. 2018 Aug 14;13(8):e0201546. doi: 10.1371/journal.pone.0201546 (PMC6091946; doi:10.1371/journal.pone.0201546)
Supplement: S5 Table — (DOCX) [file pone.0201546.s006.docx]

S5 Table Comparisons of SNV and Indel number 11 EBV+DLBCL in age and pathological subtype

| **Variables** | **groups** | **SNV** | | **Indel** | | **Nonsynonymous**  **-SNV** | | **Synonymous**  **-SNV** | |
| --- | --- | --- | --- | --- | --- | --- | --- | --- | --- |
|  |  | **mean ±SD** | ***P*-value** | **mean ±SD** | ***P*-value** | **mean ±SD** | ***P*-value** | **mean ±SD** | ***P*-value** |
| **Pathological**  **subtype** | **PL (n = 4)** | **3628±2508** | **0.415** | **1830 ±748.29** | **0.523** | **169.50±51.93** | **0.783** | **69.75 ±13.67** | **0.647** |
|  | **LCL (n = 7)** | **2244.3 ±790.2** |  | **1578.4 ±1453** |  | **166.43 ±53.12** |  | **86.71 ±34.70** |  |
| **Age** | <50 years **old (n=3)** | **2418±109.5** | **0.921** | **1279.3 ±189.6** | **0.621** | **186.33±70.50** | **0.766** | **79.67±21.22** | **0.921** |
|  | **≥50 years old（n=8）** | **2871±1964** |  | **1816.4 ±1405** |  | **160.50±44.16** |  | **80.88 ±32.98** |  |

Abbrevations: EBV+DLBCL,EBV positive diffuse large B-cell lymphoma; PL, Polymorphous lymphoma; LCL, Large cell lymphoma; SNV, single nucleotide variants; Indel, Insertion/Deletion;

*Note.* Values are mean ± SD. *P* ≤ 0.05, non-parametric Wilcoxon rank-sum test was performed.
