# Supplementary material for: Genetic heterogeneity and mutational signature in Chinese Epstein-Barr virus-positive diffuse large B-cell lymphoma
Source: PLoS One. 2018 Aug 14;13(8):e0201546. doi: 10.1371/journal.pone.0201546 (PMC6091946; doi:10.1371/journal.pone.0201546)
Supplement: S6 Table — (DOC) [file pone.0201546.s007.doc]

| **S6 Table Validation for the most recurrent genes 11 EBV+DLBCL patients analyzed by WES** | | | | |
| --- | --- | --- | --- | --- |
| **Genes** | **Genomic locus** | **Primers** | **Sequence(5’ to 3’)** | **Length(bp)** |
| **LNP1** | **exon3** | **Forward** | **GCTAACATTGCACAGTCCTTTTATACT** | **184** |
|  |  | **Reverse** | **CCTTGAATGACCCATCCTCTGAGT** |  |
| **PRSS3** | **exon3 and exon4** | **Forward** | **GAGCACAACATCAAAGTCCTGGAG** | **238** |
|  |  | **Reverse** | **ACTCACCACCAAAGCTCAGAGT** |  |
| **MUC3A** | **Exon2** | **Forward** | **TGACCACACTCCCCACTACC** | **266** |
|  |  | **Reverse** | **GGTTGATGAAGAGAAGCTGGGAGTA** |  |
| **FADS6** | **Exon1** | **Forward** | **CTCACCACGTCCTGCACCAG** | **175** |
|  |  | **Reverse** | **AGACACACGCCCCAGTCCTC** |  |
| **TRAK1** | **Exon13 and exon14** | **Forward** | **CCTAGCTACCTCCACTCCAGTTCA** | **221** |
|  |  | **Reverse** | **GGAAAGGTGCCAAGTTTACAGGAC** |  |

**Abbreviation**: EBV+DLBCL，Epstein-Barr virus positive diffuse large B cell lymphoma. WES, whole exome sequence
