# Supplementary material for: Genetic heterogeneity and mutational signature in Chinese Epstein-Barr virus-positive diffuse large B-cell lymphoma
Source: PLoS One. 2018 Aug 14;13(8):e0201546. doi: 10.1371/journal.pone.0201546 (PMC6091946; doi:10.1371/journal.pone.0201546)
Supplement: S7 Table — (DOCX) [file pone.0201546.s008.docx]

| **S7 Table Clinicobiological features of 16 additional EBV+DLBCL patients analyzed by Sanger Sequencing** | | | | | | | | | | | |
| --- | --- | --- | --- | --- | --- | --- | --- | --- | --- | --- | --- |
| NO. | Age/  gender | Pathological subtype | Lesion sites | B-symptoms | Ann Arbor Stage | LDH  (>ULN) | **β2-Microglobulin (>ULN)** | IPI Risk | therapy | response | Status  (‡OS, month) |
| 1 | 73/F | **PL** | **Left cervical LN** | yes | **IIIB** | **yes** | **NA** | **NA** | **R-CHOP** | **CR** | NA |
| 2 | 51/M | LCL | **cervical LN+** Bilateral inguinal  LN +Brain+ retroperitoneal burl mass | yes | **IIIB** | **yes** | **NA** | **NA** | **R-EPOCH** | **CR** | NA |
| 3 | 53/M | PL | **cervical** LN+ spleen + Bilateral inguinal  LN  + **mediastinum** | yes | **IIIB** | **NA** | **yes** | **NA** | **4*CHOP+1*R-CHOP** | **PD** | DOD (3.5) |
| 4 | 57M | LCL | **left** tonsil | yes | **NA** | **NA** | **NA** | **NA** | **NA** | **NA** | **NA** |
| 5 | 9/M | LCL | **Right cervical LN** | NA | **NA** | **NA** | **NA** | **NA** | **NA** | **NA** | NA |
| 6 | 18/M | PL | **left cervical LN** | NA | **NA** | **NA** | **NA** | **NA** | **NA** | **NA** | **NA** |
| 7 | 59/F | LCL | both cervical LN +axilla LN+ left inguinal LN+ spleen+ Pleural effusion+ ascites | yes | **IVB** | **NA** | **NA** | **NA** | **CDOP** | **CR** | NA |
| 8 | 55/M | PL | cervical  LN+ **supraclavicular LN+** Pleural effusion+  **Pharynx** | NA | **nA** | **NA** | **NA** | **NA** | **EPOCH** | **NA** | **NA** |
| 9 | 83/M | LCL | multiple **LN** +spleen+ **pleuro+ peritoneum** | NA | **IVB** | **NA** | **NA** | **NA** | **mEPOCH** | **PR** | NA |
| 10 | 50/M | PL | Bone marrow+mutilple LN+spleen+tonsil+pleura  +pharynx | **NA** | **NA** | **NA** | **NA** | **NA** | **REPOCH** | **NA** | **NA** |
| 11 | 56/M | PL | right cervical LN | **NA** | **NA** | **NA** | **NA** | **NA** | NA | NA | NA |
| 12 | 64/M | LCL | mutiple LN+ duodenum | **NA** | **NA** | **NA** | **no** | **NA** | **ABVD** | **PR** | **NA** |
| 13 | 74/M | PL | right cervical LN+ right inguinal  LN | **NA** | **NA** | **NA** | **yes** | **NA** | **NA** | **NA** | **NA** |
| 14 | 56/M | PL | cervical LN+ supraclavicular LN | **NA** | **NA** | **no** | **yes** | **NA** | **ABVD** | **NA** | NA |
| 15 | 34 /M | PL | cervical LN | **NA** | **NA** | **NA** | **NA** | **NA** | **NA** | **NA** | **NA** |
| 16 | 63/F | PL | cervical LN | **no** | **III** | **NA** | **NA** | **2** | **NA** | **NA** | 2+ |

Abbreviations: F,female; M, male; PL, polymorphous lymphoma; LCL, large cell lymphoma ;LN ,lymphoma node; NA, not avilable; R-EPOCH, Doxorubicin, vincristine, etoposide;PD, progressive disease; AWD: alive with disease; DOD, died of disease ；CHOP , Cyclophosphamide, doxorubicin, vincristine and prednisone; R-CHOP, rituximab, Cyclophosphamide, doxorubicin, vincristine and prednisone; CR, complete response; IPI, International Prognostic Index; LDH, Lactate dehydrogenase; IPI, International Prognostic Index; ULN, upper level of normal; WES, whole exome sequencing. . ‡OS from sampling.
